# Synthesis Mechanism and Therapeutic Effects of Thiosulfinates and Polysulfides of Different Species of Garlic from the *Allium* Genus

**DOI:** 10.3390/pharmaceutics17040437

**Published:** 2025-03-28

**Authors:** Ana Montserrat Corona-España, Mario Alberto García-Ramírez, Ingrid Mayanin Rodríguez-Buenfil, Jorge Iván Delgado-Saucedo, Orfil González-Reynoso

**Affiliations:** 1 Department of Chemistry, University Center of Applied Sciences and Engineering, University of Guadalajara, Guadalajara 44430, Mexico; ana.corona@academicos.udg.mx; 2 Department of Electro-Photonic Engineering, University Center of Applied Sciences and Engineering, University of Guadalajara, Guadalajara 44430, Mexico; mario.garcia@academicos.udg.mx; 3 Center for Research and Assistance in Technology and Design of the State of Jalisco, A.C Southeast Sub-Headquarters, Mérida 97070, Mexico; irodriguez@ciatej.mx; 4 Department of Pharmacobiology, University Center of Applied Sciences and Engineering, University of Guadalajara, Guadalajara 44430, Mexico; jorge.delgado@academicos.udg.mx; 5 Metabolic Engineering and Bioinformatics Laboratory, Department of Chemical Engineering, University Center of Applied Sciences and Engineering, University of Guadalajara, Guadalajara 44430, Mexico

**Keywords:** thiosulfinates, polisulfides, therapeutic effects, *Allium genus*, alliinase and alternative medicine

## Abstract

The genus *Allium* contains more than 300 species where garlic, onion and leek can be found. Recent studies highlight the potential of phytochemicals present in the genus *Allium* as therapeutic agents, such as antimicrobial, antihypertensive, antioxidant and antiinflammatory, which makes it a widely studied genus and an attractive option for both the pharmaceutical and food industries. This review aims to explore the current knowledge in this field. It highlights key findings regarding the pharmacological approach on thiosulfinates and polysulfides for *Allium sativum*, *Allium ampeloprasum* and *Allium sphaerocephalon* species. Further, by integrating what has been reported in previous research, this review proposes an action mechanism for the formation of thiosulfinates and polysulfides, which will help harness the therapeutic potential of nature-inspired solutions in combating illness.

## 1. Introduction

The genus *Allium* features a broad variety of species such as *Allium sativum* (Italian garlic), *Allium ampeloprasum* (elephant garlic) and *Allium sphaerocephalon* (stork’s garlic). Those species belong to the Liliaceae family and the Allioideae subfamily, which is widely consumed internationally, occupying seventh place worldwide, where Mexico occupies fifth place as a producer [1,2]. Based on records found in Egypt that concern *Allium* species use and management, it is believed that the origin of these plants can be related to Eurasia as one of the oldest plants farmed by human beings [2]. As a general botanical description, it shows a bulbous plant 30–40 cm high featuring leaves and flowers. The leaves are long and narrow with a pendulous flower grouped in a terminal umbel whose colors range from white to violet. The fruits are bulb-shaped, known as cloves, and comprise the head. Bulbs are covered by a white membrane, which can also be purplish, pink or brown [3].

The scientific classification for *Allium* species follows—Kingdom: Plantae, Division: Magnoliophyt, Class: Liliopsida, Order: Asparagales, Family: Amaryllidaceae, Subfamily: Allioideae, Tribe: Allieae, Genus: *Allium* and species, where the most studied species of garlics correspond to *Allium sativum*, *Allium ampeloprasum* and *Allium sphaerocephalon*, together with *Allium cepa*, which is colloquially known as onion [1,4].

The growing interest in these species is due to their nutritional value and the broad variety of therapeutic properties they offer as an antioxidant, anthelmintic, antiseptic, hypoglysemic, antiinflamatory, among others. These therapeutic properties are attributed to bioactive compounds present in these plants, such as steroids, flavonoids, glycosides, saponins, tannins, alkaloids, terpenoids, and especially their high content of phenolic compounds together with sulfur compounds (alliin, allicin, ajoene, allylpropyl disulfide, diallyl trisulfide, S-allylcysteine and S-allylmercaptocysteine) [1,5,6]. The sulfur compounds can be classified depending on their structure, as thiosulfinates (diallyl sulfide, diallyl disulfide, diallyl trisulfide, diallyl tetrasulfide, etc.) and as polysulfides (aliin, allicin, s-allylmercaptoglutathione, a-allylmercaptocysteine, allyl mercaptan, allyl persulfide, dithiins, etc.) [7].

In recent years, nutraceutical preparations used in natural food supplements, as well as the bioactive properties of plant species containing alliin, have been studied. The thiosulfinates present in these species, in particular, constitute an attractive alternative to artificial chemicals in the pharmaceutical industry, given the growing demand for natural and biologically active ingredients [8].

Unlike other plants, sulfur compounds from the *Allium* genus are not available for extraction, purification, and subsequent use. It is necessary to induce its synthesis through the action of the enzyme alliinase after its release and encounter with alliin as a substrate [1,5,6,9,10].

The growing interest in sulfur compounds derived from plants, by the food, cosmetic and pharmaceutical industries, is due to the high demand for natural medicinal products and their high effectiveness [11]. However, these therapeutic molecules face significant challenges related to their quality, particularly inconsistency, lack of safety due to the instability of these compounds, and their efficacy, which is directly affected by their extraction, the presence or absence of which depends on how the enzymatic reaction mediated by the alliinase enzyme is carried out [1,10].

To ensure the safety and efficacy of these products, it is essential to have standardization methods that cover every aspect, from the extraction of the plant material to the analysis of the processing and final product, but above all, it is essential to fully understand the enzymatic reaction of the alliinase enzyme, which is still being elucidated. Therefore, this research focused on exploring the current state of the art in this field, highlighting key findings concerning the pharmacological approach toward thiosulfinates and polysulfides from the *Allium sativum, Allium ampeloprasum* and *Allium sphaerocephalon* species. By integrating what has been reported in previous research, we also propose an action mechanism for the formation of thiosulfinates and polysulfides. This was accomplished through a systematic bibliographic review using specific descriptors in databases such as Pubmed, Science Direct, SciELO, Scopus, as well as academic papers and books from 1951 to 2025. To harness the therapeutic potential of nature-inspired solutions from the *Allium* genus, we grouped together information from the various species into a single up-to-date action mechanism for the synthesis of sulfur compounds such as thiosulfinates and polysulfides.

## 2. Biochemical Garlic Composition

The micronutrient content from the genus *Allium* garlic bulbs is highly influenced by the content present in the soil where it grows. For example, 100 g of fresh garlic shows bioactive compounds and micronutrients such as organosulfur compounds, volatile sulfur compounds, proteins, prostaglandins, fructans, vitamins, polyphenols, fatty acids and essential oils in the proportions listed in Table 1 [3,12,13]. Some of the most abundant sugars reported among those plants are heterosides, glycosides, reduced sugars, fructans and galactosides, which work generally as plant storage, as an energy source for protection against herbivores, or as a structural component [3].

Nowadays, the genus *Allium* remains as one of the most studied medicinal plants due to its abundance in therapeutic properties, with secondary metabolites like flavonoids, sulfur compounds, pyruvic acid, tannins, anthocyanins and saponins [14]. The *Allium sativum* species contains around 33 compounds derived from sulfur, such as alliin, allicin, ajoene, allylpropyl disulfide, diallyl trisulfide, S-allylcysteine and S-allylmercaptocysteine. Those compounds can be divided into two large groups according to solubility. The fat-soluble compounds include allyl sulfur, diallyl disulfide (DADS) and diallyl trisulfide (DATS), followed by those that are water-soluble, e.g., γ-glutamyl S-allylcysteine, S-allylcysteine (SAC) and S-allylmercaptocysteine (SAMC), as listed in Table 2 [15].

## 3. Thiosulfinate and Polysulfide Synthesis Mechanism

Alliin lyase (aliin alkyl-sulfenate-lyase, S-alk(en)yl-L-cysteine sulfoxide lyase, alliinase, EC 4.4.1.4) belongs to the glycoproteins family [10,18,19]. It is estimated to have a carbohydrate content of 5.5% in garlic species and a 4.6% in onion species, with the molecular mass of the enzyme molecule reported at 140, 150 and 200 kDa [20].

**Figure 1:**
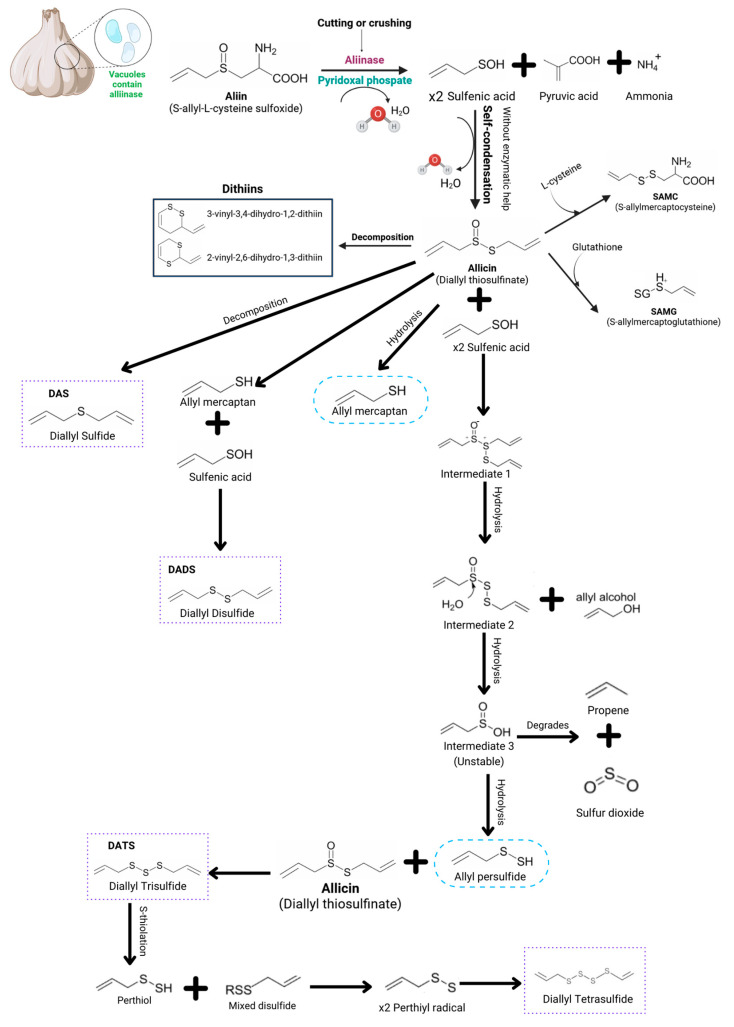
Schematic diagram that features the alliinase enzyme action mechanism for the formation of thiosulfinates and polysulfides. Here, the diagram shows that once alliinase is released from the vacuoles, it encounters alliin, by initiating a cleavage in the carbon–sulfur bond within amino acids. It leads to the formation of an unstable complex that undergoes dehydration in the presence of pyridoxal phosphate that results in three intermediates: sulfenic acid, pyruvic acid and ammonia. This process triggers a series of reactions from allicin, ultimately transforming into thiosulfinates and polysulfides such as diallyl sulfide, diallyl disulfide, diallyl trisulfide, diallyl tetrasulfide, γ-glutamyl, and S-allylcysteine derivatives like S-allylcysteine (SAC) and S-allylmercaptocysteine (SAMC), along with ajoenes [3,7,9,18,19,20,23,24,25,26,27,28,29,30].

Alliinase represents up to 10 percent of the protein in garlic cloves and is housed in the vacuoles present in the garlic clove cells [21,22]. It has been characterized from several plant tissues, including *Allium sativum* cloves and *Acacia farnesiana* and *Brassica oleracea* buds. Related enzymes have also been identified in bacteria and in fungi [20]. These enzymes are usually separated from the substrate in vivo, as it is located in the cytoplasm, while the enzyme in the vacuole is released through mechanical stress by grinding, cutting or crushing [19,20,21,22]. The backbone reaction is depicted in Figure 1. Alliinase is a pyridoxal phosphate (Pxy-P)-dependent enzyme as a cofactor [18]. Once the alliinase is released, it meets alliin, an abundant compound in garlic species, which behaves as a substrate during the reaction. It starts with the carbon–sulfur bond rupturing in the amino acid side by a beta-elimination, which is spontaneously hydrolyzed to yield three complex unstable intermediates: pyruvate, ammonia and a sulfur-containing product [2,9,18,19,21].

Sulfur-containing metabolites, such as thiosulfinates, thiols, disulfides, etc., are secondarily produced from sulfenic acid through non-enzymatic reactions [18]. Figure 1 shows a reaction series that is triggered by allicin, culminating in the formation of thiosulfinates and polysulfides, i.e., after consuming garlic, an in vivo reaction occurs due to the allicin interaction with amino acids such as glutathione and cysteine. It leads to sulfur compounds such as S-allylmercaptocysteine and S-allylmercaptoglutathione, respectively, through an S-thiolation. The subsequent 2-propenesulfenic acid addition can blueprint the synthesis of ajoenes [2,15,22].

Alternatively, through allicin hydrolysis reactions, allyl mercaptan and allyl persulfide can be formed. However, the allicin molecules and sulfenic acid interaction through hydrolysis reactions point to the formation of several intermediates that culminate in thiosulfinate formation, such as diallyl trisulfide and diallyl tetrasulfide, which are the first stable sulfur-containing products of the reaction, also known as alkyl alkane thiosulfinates [2,19].

As described, the sulfur compounds enter a cascade of further reactions to produce alkyl disulfides and polysulfides, like vinyl dithiins and ajoene, depending on the concentration, temperature and pH [23,24].

Alliinase has been previously isolated and purified. However, complications have been reported concerning its molecular mass and the biochemical characteristics for its use [10,23]. For this reason, it is important to consider its working conditions, as it is activated at a pH of 4.5 to 9 and can be irreversibly inactivated at an acidic pH of 1.5 to 3. It is a very sensitive enzyme to temperature; its activity decreases considerably at 42 °C and it can denature at temperatures greater than 60 °C [23].

This is important because the chemical composition of the preparations obtained from garlic extractions depends first on the enzymatic reaction, but also on the extraction conditions, such as temperature, time and solvent polarity. The biological activities that the sulfur compounds show depend on many factors, including the origin of the raw material, the conditions in which the enzymatic condition took place to decompose the organosulfur compounds, and on the methods of isolation [17,31].

Concerning the use of sulfur compounds as therapeutic molecules, the importance of the synthesis reaction and extraction methods needs to be emphasized in order to understand how the compounds migrate from the intracellular matrix in which they were formed.

## 4. Extraction and Analysis of Sulfur Compounds

A broad variety of techniques exist for extracting sulfur compounds from the genus *Allium* by both traditional and innovative approaches. Traditional methods use either polar or non-polar solvents by allowing the desired compounds to transfer into the solvent through diffusion or mass transfer mechanisms. Conventional techniques include maceration, steam distillation and mechanical extraction. In contrast, unconventional methods aim to enhance conventional processes by reducing energy consumption by improving yields and minimizing solvent usage. These innovative approaches include ultrasound-assisted extraction (UAE), microwave-assisted extraction (MAE), high-speed shear homogenization extraction (HSHE), ultrasound-assisted enzymatic extraction (UAEE) and microwave ultrasound-assisted extraction (UMAE), among others [17,32,33]. There is a broad variety of techniques for sulfur compound extraction from the genus Allium, by both traditional and innovative approaches. Traditional methods use either polar or non-polar solvents, facilitating the transfer of the desired compounds into the solvent through simple diffusion or mass transfer mechanisms. Examples of conventional techniques include maceration, steam distillation and Soxhlet [25,26].

A key method to facilitate the release of the beneficial compounds once a solvent is added involves maceration, where garlic is either crushed or sliced. The solvent selection is based on polarity with a preference for polar or moderately polar solvents. Some of the most common ones used for garlic extraction include methanol, a variety of ethanol strengths (96, 95, 75 and 70 °C), hexane, acetone and hydroalcoholic blends [7]. Once the garlic and solvent are combined, the container is sealed and stored in a place with controlled temperature and away from direct sunlight. The resting period varies according to the desired compounds, ranging from a few days to a few weeks (2–4 weeks). Through this period of time, the mixture should be occasionally gently stirred to help the extraction process. After the soaking period, the mixture is filtered by using cheesecloth, a fine mesh strainer or a coffee filter to eliminate solid particles before being transferred to a dark glass bottle and sealed [16,27]. Another conventional method is steam distillation. It is ideal for essential oil extraction. According to the *European Pharmacopoeia 7.0* (2019) and the study carried out by Tran et al. in 2023, the samples should be finely milled and homogenized before placing them in a flask with 1 mL of xylene added to the oil collection tube. The flask liquid is usually a NaCl solution that is heated until reaching the boiling point, and the distillation rate is set to a range of 2 to 3 mL/min. It is recommended to dehydrate the oil by using sodium sulfate for subsequent storage in a brown bottle at 5 °C, in order to calculate the yield [28]. Soxhlet is a solid–liquid extraction, also considered a conventional method, that is mainly used to isolate lipid components in a non-polar solvent such as petroleum ether and hexane. It is recommended for samples where the free-fat fraction is sought [29].

Despite the disadvantages offered regarding time and solvent consumption, those methods are still in use today due to the simplicity in implementing the methodology and the inexpensive apparatus needed for key extraction rates [30]. For that reason, unconventional methods mainly focus on enhancing conventional processes by reducing energy consumption, improving yields and minimizing solvent usage [33]. Those innovative approaches are mentioned elsewhere [23,33].

Ultrasonic-assisted extraction uses ultrasonic wave energy within the solvent during the extraction, producing cavitation by accelerating the solute and heat transfer diffusion, which improves the extraction efficiency [23]. Microwave–ultrasound-assisted extraction generates heat due to the polar compound’s interaction, the organic components in the plant matrix are released by mechanisms such as ionic conduction or dipole rotation. The process does improve the extraction yield [23,34]. Alternately, enzyme-assisted extraction benefits are due to the enzyme’s hydrolytic action on the cell wall components, such as cellulose, α-amylase and pectinase, making easier for the metabolites of interest to be released through the secondary metabolism of plants [23].

The components extracted from the methods explained elsewhere are complex and feature a broad variety of natural products. The reason for this is that further separation and purification processes are required to obtain the active fraction or the pure natural metabolites. The separation depends on important physical or chemical characteristics [23]. The determination and quantification of sulfur compounds are a difficult task due to the difficulty of allicin reference compound extraction process. It is mainly because of the great instability that these metabolites present. However, favorable results have been obtained by using HPLC analysis [25,26].

## 5. Genus *Allium* Therapeutic Effects

### 5.1. Antimicrobial and Antiviral Properties

Species such as *Allium sphaerocephalon* contain a large amount of cysteine sulfoxides by including alliin, methiin and isoallin. It has been reported elsewhere in vitro tests that these inhibit *E. coli*, *P. aeruginosa*, *S. aureus*, *B. subtilis* and *C. albicans* biofilm formation, with ethanolic extracts obtaining favorable results [12,35,36]. However, allicin, itself, is effective against Gram-positive and Gram-negative bacteria, but both ajoenes and diallyl trisulfide can present similar effects [3]. Studies on the inhibition of bacteria such as *E. coli* and *L. innocua* using aqueous extracts of *Allium sativum* (Italian garlic) have shown it to be effective at concentrations of 0.25 and 0.125 g garlic/mL of water for those microorganisms, respectively [3,16,37].

Allicin shows key antibacterial and antifungal properties while ajoene shows promising results as an antiviral agent [38]. There has been limited research on garlic’s antiviral capabilities. Studies that use garlic extracts rich in allicin, ajoene and diallyl trisulfide have shown effectiveness against influenza A and B, cytomegalovirus, rhinovirus, HIV, herpes virus 1 and 2, viral pneumonia and rotavirus [39]. Furthermore, recent important publications suggest that those active compounds can mitigate the inflammation from dengue virus infections by an action mechanism involving oxidative stress response modulation and interferon gamma, which increase lymphocytes proliferation, macrophages and natural killer cells, thus positively impacting the immune system [3,40].

### 5.2. Anticancer Properties

Allicin and thiosulfinates (DADS, DATS and DAS) in combating cancer can be examined through a broad variety of angles by including the ability to prevent cell proliferation and promote programmed cell death. For the anticancer and antioxidant properties of allicin, Ravindra et al. performed research in 2023 on zebrafish. The analysis revealed that embryos treated with allicin features a reduction in pH3-positive cells compared to the control group [41]. The results suggest a suppressive effect on cell proliferation. The effect was observed even in conditions where TP53, a tumor suppressor gene, was mutated, indicating involvement in a TP53-independent apoptotic pathway [42]. Research on garlic extraction rich in DADS and DAS has shown to be effective in breast and bladder cancer adducts [43]. One of the first steps in chemically induced carcinogenesis is the formation of DNA adducts, which results from the free radical elimination mechanism. It occurs after increases or modulation in glutathione levels and in the related enzymes such as glutathione- s-transferases and catalases [3,10]. The glutathione-S-transferase enzymatic activity modulation is another key property attributed to sulfur compounds. It is important due to its crucial role in the detoxification of carcinogens and cytochromes P450 (CYP) [15]. Studies where methanolic extracts of *Allium sativum* were used on *Escherichia coli*, showed that the action mechanisms of sulfur compounds include the inhibition of mutagenesis caused by 4-nitroquinoline-1-oxide [23].

Research by Upadhyay et al. in 2025 showed that treatment with crude garlic extract from *Allium sativum* ethanolic extract significantly suppressed the growth of human MDA-MB-231 cells compared to breast cancer cells MCF-7 [43]. Another study mentions that garlic hinders the development of lung, hepatic, gastric and prostate cancer by arresting the cell cycle and inducing apoptosis [44].

### 5.3. Antihypertensive Properties

**Figure 2:**
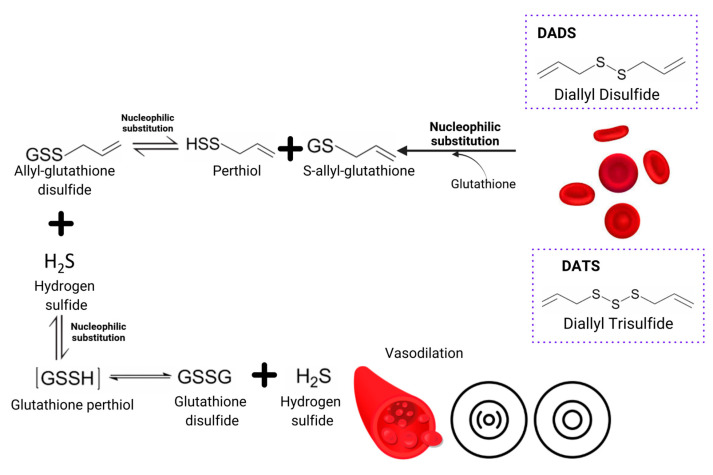
Schematic diagram that features the DADS and DATS conversion into hydrogen sulfide by the erythrocyte. It is a phenomenon known as the endothelium-derived relaxing factor mechanism, which ends in vasodilation [2,10].

Garlic cardioprotective activities occur due to the action mechanism related to enzyme inhibition that actively participates during cholesterol synthesis, such as hydroxymethylglutaryl- coenzyme A reductase (HMG-CoA) and lanolesterol-14-dimethylase. Allicin acts over lipid metabolism and insulin resistance by increasing the AMP-activated protein kinase (AMPK) phosphorylation and decreasing the expression of sterol regulatory element binding protein 1 (SREBP-1) and SREBP-226 [44]. Improvements in LDL oxidation by inhibiting cholesterol synthesis in the liver have also been observed [3]. Allicin and ajoene inhibit the cyclooxygenase and lipoxygenase that exert effects on platelets ADP, collagen and fibrinogen receptors, contributing to an antiaggregant effect. Those act as powerful vasodilators by increasing the nitric oxide synthetase levels, known as the endothelium-derived relaxing factor. The mechanism is described in Figure 2. Ajoene can reduce calcium levels in smooth muscle cells, causing vasodilation [3,26,27].

### 5.4. Antiinflammatory and Antioxidant Properties

When discussing inflammatory responses, it is important to consider it as an immune system response to aggression, which gives rise to a process that features several stages such as mediating-substances release (cytokines IL-1, IL-6, IL-8 and TNF-α) as well as immune cells and molecules that are activated and, as a consequence, release excess inflammatory mediators such as prostaglandin E2 (PGE2), nitric oxide (NO) and proinflammatory cytokines (IL-6) [17,45,46,47]. Thiosulfinates have demonstrated massive capabilities against chronic inflammation and oxidative stress through immune cell modulation, i.e., nuclear factor κβ and enzymes such as glycogen synthase kinase (GSK)-3β [14,17]. Further, it leads to NFκβ pathway suppression, thereby preventing prolonged inflammation, cell damage and tissue injury [27].

*Allium* species, particularly garlic, possess antiinflammatory properties than might be highly beneficial for the female reproductive system, regardless of endometriosis and pelvic inflammatory disease. Additionally, sulfur compounds serve as antioxidants by offering protection against oxidative stress and damage caused by free radicals. The characteristic mainly given by allicin reduces reproductive disorder risks. Reactive oxygen species present in garlic extracts are important in signaling processes. Concerning infertility problems, reactive oxygen species play a key role over the excessive production of oxidants [14,27].

Specimens of *Allium cepa* and *Allium sativum* leaves and bulbs show that the *Allium* species antioxidant power comes primarily from sulfur compounds and bioactive components such as polyphenols, dietary fiber and microelements [14,28]. The antioxidant function is provided through the formation of uric acid and assisted by the enzyme xanthine oxidase, which binds to the molybdopterin (Mo-Pt) domain to prevent the enzyme activity [14]. In contrast, recent investigations demonstrate that phenolic compounds can cross the blood–brain barrier by creating protective effects on neuronal cells that, together with organosulfur compounds, can reduce the neuroinflammatory microglial cell response through the mediation of Nrf2, which is related to oxidative stress [41,48].

### 5.5. Dosage and Contraindications

Records have been found that describe *Allium*’s use since ancient times, due to numerous properties it offers as an antimicrobial, antihypertensive, antiinflammatory, antiplatelet agent, among others. The effective garlic doses are still unknown. For that reason, it is generally considered that for the average adult, a recommended daily dose of *Allium sativum* is 4 g of garlic, 300 mg of encapsulated pulverized garlic or 7.2 g of aged garlic extract [49]. The genus *Allium* possesses contraindications; it is well known that the anticoagulant action from drugs such as heparin or warfarin favor the appearance of hemorrhages [3]. Therefore, it is recommended to suspend the consumption of any product derived from the genus *Allium* at least 10 days before any surgical intervention involving bleeding prevention [49]. Moreover, it is important to avoid the concomitant medications use such as non-steroidal antiinflammatory drugs (NSAIDs) and drugs that inhibit hepatic metabolism (cimetidine, ciprofloxacin, clarithromycin, diltiazem, erythromycin, fluoroxetine, ketoconazole, paroxetine and ritonavir), as it interacts with alprazolam, amitriptyline, carbamazepine, cisapride, clozapine, corticosteroids, cyclosporine, diazepam, imipramine, desipramine, phenytoin and propranolol [12,50]. Nevertheless, garlic is considered non-toxic but it might cause bad breath or body odor [3]. In people sensitive to the compounds present in garlic, it can cause abdominal pain, fullness feelings, nausea and flatulence. During pregnancy and lactation, the use of garlic for therapeutic purposes can be dangerous since it is associated with abortive effects, alterations in menstrual cycles and in uterus activity [49]. It is therefore recommended to have medical assistance for the use of these natural products.

## 6. Therapeutic Advances and Future Prospects of Sulfur Compounds (Thiosulfinates and Polysulfides) from the *Allium* genus

In recent years, sulfur compounds derived from the genus *Allium*, such as thiosulfinates and polysulfides, have captured the attention of the scientific community due to their promising therapeutic properties, as described elsewhere. Several studies have evaluated their effects in important areas of health, revealing encouraging results. However, the variability in the composition derived from the enzymatic reaction of the synthesis of these compounds and their stability has posed challenges in their clinical application. In this brief summary, we focus on the most relevant studies that have investigated the therapeutic effects of *Allium* thiosulfinates and polysulfides, and their possible future applications in medicine. Through a review of these works, we provide a comprehensive view of the progress made to date and the future prospects for the use of these compounds to improve human health.

Scientific studies have shown that garlic and its related compounds, especially cysteine sulfoxide, S-allyl cysteine sulfoxide, allyl propyl disulfide and allicin, can inhibit protein tyrosine phosphatase 1B (PTP1B) in the insulin signaling pathway, thus exhibiting an antidiabetic effect through the reduction in glucose level by stopping the insulin activation produced by the liver, isolation of insulin from the bonded forms, and facilitating the production of insulin from β-cells that increase the cell sensitivity to insulin. It is important to note that the negative regulatory effect of protein tyrosine phosphatase 1B (PTP1B) in the insulin signaling pathway makes it a promising therapeutic target for Type II diabetes mellitus treatment [51].

Studies related to its antibacterial potential carried out with essential oils of *Allium sativum* through both in vitro and in silico approaches demonstrated robust activity against methicillin-resistant *Staphylococcus aureus* with an inhibition zone of 22 mm and a minimum inhibitory concentration of 0.75 mg/mL [52]. In addition to this therapeutic property as an antimicrobial, recent studies seek to develop an approach to convert natural organosulfur compounds into nanometer-sized iron sulfides (nFeS), in order to enhance antibacterial activity on pathogenic bacteria with drug resistance or associated with biofilms [53].

Regarding inflammation, interesting results have been obtained. Several studies have shown that garlic has antiinflammatory effects, especially when used in extract form. This, in turn, shows a relationship with the immune system, demonstrating its strong activity in the modulation and induction of cytokines, promoting resistance to pathogenicity and the activation of phagocytic functions, such as immunoglobulins and macrophages. These garlic extracts have the ability to restrict or downregulate the viral antagonism strategy, inhibiting the competition of the NS1 protein, reducing influenza virus replication in cells. Alternately, the administration of s-allyl-cysteine mercapto-l-cysteine (SAC) has demonstrated its ability to suppress inflammatory events in asthma, specifically downregulating TH2 cytokines, which can attenuate neuroinflammatory responses in microglial cells by modulating Nrf2-mediated signaling and other pathways related to oxidative stress [54,55,56,57,58,59]. From these anti-inflammatory mechanisms, some research has sought to take advantage of these compounds, seeking a way to integrate them into current therapeutics and technologies. Within these proposals, we find the development of models of acrylamide polymerization under conditions of aqueous drying, and 1,3-dimethylimidazolium dimethyl phosphate for the formation of phosphorylated linear sulfur oligomers for its use in this field [60].

Additionally, the natural phytochemicals present in garlic have gained relevance due to their ability to inhibit the formation of advanced glycation end products and, in parallel, mitigate the damage caused by free radicals by modulating the AGE-RAGE signaling pathway, reducing oxidative stress, inflammation and endothelial dysfunction, crucial factors in microvascular complications associated with diabetes. This has been demonstrated in studies on mice infected with *Trypanosoma evansi*, which significantly improved their liver structure after being treated with *Allium sativum* extracts. Furthermore, parasitemia was suppressed by 91.5% when following the same mechanism of inhibition of advanced glycation end products [57,58,59,60].

Molecules of natural origin have been used in traditional medicine for centuries as an invaluable source of bioactive compounds with therapeutic properties. These compounds have prevailed due to their high effectiveness and are still in use to-date. The future perspective is that these areas of knowledge will expand and these ancestral therapies will be integrated with new technologies such as pharmacogenomics, the use of bioinformatics tools, and materials or polymers, which will be applied to develop safe and effective extended-release therapies.

## 7. Summary and Conclusions

The alliinase enzyme belongs to the glycoprotein’s family housed in the vacuoles present in the garlic clove cells, and it is released through mechanical stress. Once alliinase is released from the vacuoles, it encounters alliin, by initiating a cleavage in the carbon–sulfur bond within amino acids. It leads to the formation of an unstable complex that undergoes dehydration in the presence of pyridoxal phosphate, which results in three intermediates: sulfenic acid, pyruvic acid and ammonia. This process triggers a reactions series from allicin, ultimately transforming into thiosulfinates and polysulfides such as diallyl sulfide, diallyl disulfide, diallyl trisulfide, diallyl tetrasulfide, γ-glutamyl and S-allylcysteine derivatives like S-allylcysteine (SAC) and S-allylmercaptocysteine (SAMC), along with ajoenes.

Unlike the base information that was available to describe the enzymatic reaction, the advances that have been made in recent years allow us to understand and highlight the following critical points of the reaction: 

The first point is to understand that there are two reactions that can occur once allicin enters into a biological system or comes into contact with amino acids such as L-cystine and glutathione, forming S-allylmercaptocysteine (SAMC) and S-allylmercaptoglutathione (SAMG). Alternately, when the literature mentions that the formation of these sulfur compounds is due to decomposition, it refers to decomposition by thermal conditions, since these are very thermosensitive compounds. Finally, the formation of sulfur compounds occurs gradually and consecutively, where the formation of compound “A” gives rise to a new reaction for the formation of compound “B”, where the compound “A” can act as an intermediary for the synthesis of compound “B” or “C” or “D”, depending on the route followed. Making this mechanism a self-feeding route until the substrate of the intracellular matrix is exhausted, it reaches product saturation depending on the path taken by the reaction. That is, the action mechanism of the enzyme alliinase presents a multi-substrate reaction behavior, since from a base substrate, in this case alliin, allicin is formed, which subsequently functions as a substrate for the formation of another thiosulfinate or polysulfide that will subsequently function as a substrate for the following reaction, giving rise to multiple products. In turn, the enzyme forms intermediate complexes, which are described as temporary with the substrates and the products before releasing them, making this a complex enzymatic mechanism, whose products are molecules with high therapeutic power.

However, to make the best use of these benefits, it is vitally important to have a complete overview of the enzymatic reaction responsible for the synthesis of these compounds. Previously, the first reaction that gives rise to allicin from alliin was well known and, generally, most of the literature describes that allicin passes to other sulfur compounds as a consequence of decomposition, leaving aside the reactions involved in obtaining these products. Over the years, discoveries have been made that gradually revealed and completed the puzzle of this complex but fascinating enzymatic reaction of the alliinase enzyme. Therefore, one of the main purposes of this research was to integrate and propose a single reaction mechanism for the formation of thiosulfinates and polysulfides, using the knowledge available to-date.

Sulfur compounds, products of the decomposition of alliin, by the action of the enzyme alliinase, into allicin, thiosulfinates and polysulfides, are becoming increasingly important due to their multiple therapeutic properties that they offer, ranging from antimicrobial, anticancer, antihypertensive, antiviral, antioxidant, antiinflammatory to immunomodulatory effects.

More research is needed on optimizing extraction methodologies, whose objective is to obtain better yields with less ecological impact, to ensure both the quality of the product and the health of the user. The integration of new technologies with ancestral knowledge such as herbal medicine has become highly effective, presenting a great area of opportunity for their use in the health sciences—for people with metabolic limitations who are looking for quality alternatives, without toxicological risk and that provide a broad spectrum of benefits, as is the case of products from the *Allium genus*.

## Figures and Tables

**Table 1:** Bioactive compounds and micronutrients content for the genus *Allium* [3,12,13].

Analysis	Amount
Water	58.58 g
Energy	119–149 kcal
Protein	4.30–6.36 g
Total lipids	0.23–0.5 g
Carbohydrates	24.30–33.06 g
Dietary fiber	1.20–2.1 g
Total sugars	1–2.21 g
Vitamin C	14–31.2 mg
Vitamin B1	0.02–0.2 mg
Vitamin B2	0.11–1.02 mg
Niacin	0.60–0.7 mg
Vitamin B6	0.32–1.235 mg
Vitamin A	0.16 mg
Vitamin E	0.01–0.08 mg
Vitamin K	1.4–1.7 µg
Folate	3 µg
Calcium	17.80–181 mg
Iron	1.2–1.7 mg
Magnesium	24.10–25 mg
Phosphorus	134–153 mg
Potassium	401–446 mg
Sodium	17–19 mg
Zinc	1.10–1.16 mg
Iodine	4.70 mg

**Table 2:** Solubility and stability of the sulfur compounds [15,16,17].

Solubility	Sulfur Compound	Stability	Content Reported in the Literature
In water	s-allyl-cysteine	Very stable	19–1736.2 mg/g
	s-allyl-mercaptocysteine	Stable	
	s-methylcysteine	Stable	
	*γ*-glutamyl-cysteine	Stable	
In oil	Allicin	Very unstable	2.5–4.5 g
	Aliin	Stable	
	Diallyl sulfide	Unstable	
	Diallyl disulfide	Unstable	
	Diallyl trisulfide	Unstable	0.3–10 mg/kg
	Ajoene	Very stable	0.5–0.10 g

## Abbreviations

DADS	Diallyl disulfide
DATS	Diallyl trisulfide
SAC	S-allylcysteine
SAMC	S-allylmercaptocysteine
UAE	Ultrasound-assisted extraction
MAE	Microwave- assisted extraction
HSHE	High-speed shear homogenization extraction
UAEE	Ultrasound-assisted enzymatic extraction
UMAE	Microwave ultrasound-assisted extraction
pH3	Phospho-histone 3
TP53	Tumor suppressor gene
CYP	Cytochrome P450
HMG-CoA	Hydroxymethylglutaryl- coenzyme A reductase
AMPK	AMP-activated protein kinase
SREBP-1	Sterol regulatory element binding protein 1
SREBP-226	Sterol regulatory element binding protein 226
LDL	Low-density lipoproteins
ADP	Adenosine diphosphate
IL-1	Interleukin-1
IL-6	Interleukin-6
IL-8	Interleukin-8
TNF-α	Tumor necrosis factor alpha
E2	Prostaglandin E2
NO	Nitric oxide
(GSK)-3β	Glycogen synthase kinase
Mo-Pt	Molybdopterin
Nrf2	Nuclear factor erythroid type 2
NSAIDs	Non-steroidal anti-inflammatory drugs
PTP1B	Protein tyrosine phosphatase 1B
MDA-MB-231	Epithelial adenocarcinoma
MCF-7 cells	Human breast cancer cell line
